# Clinical outcomes of immunoglobulin use in solid organ transplant recipients: protocol for a systematic review and meta-analysis

**DOI:** 10.1186/s13643-015-0156-6

**Published:** 2015-11-19

**Authors:** Juthaporn Cowan, Brian Hutton, Nicholas Fergusson, Alexandria Bennett, Jason Tay, D. William Cameron, Greg A. Knoll

**Affiliations:** Division of Infectious Diseases, Department of Medicine, University of Ottawa, Ottawa, Canada; Clinical Epidemiology Program, Ottawa Hospital Research Institute, Ottawa, Canada; Blood and Marrow Transplant Program, The Ottawa Hospital, Ottawa, Canada; Renal Transplantation, Division of Nephrology, Department of Medicine, University of Ottawa, Ottawa, Canada

**Keywords:** Immunoglobulin, Hypogammaglobulinemia, Solid organ transplantation, Systematic review

## Abstract

**Background:**

Transplantation improves survival and the quality of life of patients with end-stage organ failure. Infection, due to surgical issues, host factors such as diabetes, immunosuppression, and hypogammaglobulinemia, is a major post-transplant complication. Clinical outcomes of prophylaxis or treatment of hypogammaglobulinemia in solid organ transplant recipients are not well established and are in need of further study.

**Methods/design:**

We will conduct a systematic review of studies investigating clinically relevant outcomes of immunoglobulin use either as prophylaxis or treatment of hypogammaglobulinemia after solid organ transplantation. Both randomized and non-randomized studies (excluding case reports and case series of less than 20 subjects) will be included. Outcomes of interest will include the overall rate of infection, hospital admission, hospital length of stay, intensive care unit admission, 1-year all-cause mortality, incidence of acute organ rejection, allograft survival within 1 year, and adverse events. We will search MEDLINE, Embase, the Cochrane Central Register of Controlled Trials, Transplant library, and the International Clinical Trials Registry Platform for randomized and non-randomized studies on adult solid organ transplant patients who received prophylactic immunoglobulin or immunoglobulin treatment. Two reviewers will conduct all screening and data collection independently. We will assess study level of risk of bias using the Cochrane Risk of Bias Assessment Tool for randomized controlled trials and for non-randomized studies. If meta-analysis of outcome data is deemed appropriate, we will use random effects models to combine data for continuous and dichotomous measures.

**Discussion:**

The results of this systematic review may inform guideline development for measuring immunoglobulin level and use of immunoglobulin in solid organ transplant patients and highlight areas for further research.

**Systematic review registration:**

PROSPERO CRD42015017620

**Electronic supplementary material:**

The online version of this article (doi:10.1186/s13643-015-0156-6) contains supplementary material, which is available to authorized users.

## Background

Hypogammaglobulinemia (gamma-immunoglobulin (IgG) <700 mg/dL or 7 g/L) is a complication of solid organ transplantation (SOT) that occurs after immunosuppressive therapy or treatment of organ rejection [1]. The reported incidence of hypogammaglobulinemia among kidney transplant recipients is between 40 and 60 % [2–5]. This is similar to the overall rate of hypogammaglobulinemia in all SOT recipients [4]. The rate, duration, and severity of hypogammaglobulinemia are further modified by a variety of additional factors including host and donor characteristics, presence of graft rejection, and its accompanying use of immunosuppressive therapy [6–8].

Immunosuppression is vital in transplantation; however, it also increases risk of infection. Hypogammaglobulinemia is associated with an increased rate of bacterial infection as compared to transplant recipients without hypogammaglobulinemia (15 vs 5 %) [9]. Post-transplant hypogammaglobulinemia is an independent risk factor for overall (hazard ratio (HR) 2.03; 95 % confidence interval (CI) 1.39–2.96) and bacterial infection (HR 2.68; 95 % CI 1.66–4.34) in kidney transplant recipients [3]. Hypogammaglobulinemia is also an independent risk factor for *Clostridium difficile*-associated colitis in heart transplantation recipients [10]. Severe hypogammaglobulinemia (IgG <4 g/L) is also a risk factor for death following SOT [4]. It is not known whether the increased risk of mortality is solely related to severe infection as a result of hypogammaglobulinemia in this population.

Intravenous immunoglobulin (IVIG) is prepared from pooled plasma involving thousands of healthy blood donors. The large donor pool ensures a diversity of antibody specificities to a wide spectrum of antigens and microbial pathogens [11]. The use of IVIG or any form of immunoglobulin in hypogammaglobulinemia after solid organ transplantation has not been well explored. A retrospective study looking at IVIG therapy and survival post-transplantation has been recently published [12]. This study showed that increasing immunoglobulin levels with IVIG did not have any impact on survival. However, the study was limited by its retrospective design and small sample size.

IVIG has been used in highly sensitized kidney transplant recipients to permit successful transplantation and reduce the risk of antibody-mediated rejection [13]. IVIG is thought to be effective in this setting by blocking complement activation, neutralizing donor-specific antibodies via anti-idiotypic HLA antibodies, and downregulating HLA antibody production by donor-specific B-cells.

To date, clinical outcomes related to the prophylactic or therapeutic use of immunoglobulin in SOT recipients have not been analyzed. The planned systematic review will address this knowledge gap in order to establish the current state of the evidence on this topic.

### Research objectives

We will conduct a systematic review to evaluate clinical outcomes associated with the use of immunoglobulin after solid organ transplantation.

## Methods

### Search strategy

A comprehensive electronic search will be conducted in MEDLINE (1950 to September 2015), Embase (1950 to September 2015), and the Cochrane Central Register of Controlled Trials (September 2015). The assistance of a medical librarian experienced in systematic reviews will be used in designing the search strategy. A structured search strategy will be based on controlled vocabulary and relevant key terms and will be broad to prioritize sensitivity (Additional file 1). Language of publication will not be restricted, and the draft search will be peer reviewed by a second medical librarian according to PRESS criteria [14]. The references of included articles and existing reviews will be scanned for additional studies that were not identified by the search. Transplant library search (www.transplantevidence.com) will be performed in order to identify studies that were published as conference proceedings or only in abstract forms. We will search the International Clinical Trials Registry Platform (www.who.int/ictrp/en/) to identify incomplete or unreported studies.

### Study screening and inclusion

Using the results of our comprehensive search strategy, we will obtain title and abstract from all references. First titles and abstracts (stage 1) and then full-texts of potentially relevant studies (stage 2) will be screened by two reviewers independently following short pilot exercises to identify and address any inconsistencies in the application of screening criteria. The inclusion and exclusion criteria used for each stage of screening are outlined below. If no abstract is available for a given citation, the full-text will be obtained unless the article can be confidently excluded by its title alone. In general, if there is any doubt as to whether a study should be excluded, the study will proceed to the full-text screen to reduce the likelihood of excluding a relevant study. A third party will reconcile any disagreements if discussion between the two reviewers cannot determine the article’s inclusion status. The process of study selection will be summarized using a PRISMA flow diagram [15].

### Study eligibility criteria

Studies will be selected according to the criteria outlined in Table 1.

**Table 1:** PICOS breakdown of study eligibility criteria

Category	Description of criteria
Population	Adults undergoing solid organ transplant (including the heart, lung, liver, kidney, intestine, and pancreas)
Intervention	Polyvalent immunoglobulin prophylaxis or polyvalent immunoglobulin treatment without restriction to dosage, frequency, timing, and route of administration
Comparator(s)	Placebo or no treatment. Patients may receive antimicrobial prophylaxis, which should be the same in both intervention and control arms
Outcome(s)	Primary outcome: 1) Overall rate of infection 2) Rate of infection based on severity: non-severe infection, severe infection, and serious/fatal infection
	Secondary outcomes: 1) Hospital admission, initial length of stay, and number of re-admissions in the first year after transplantation; 2) ICU admission; 3) 1-year all-cause mortality; 4) Incidence of acute rejection; 5) Allograft survival at 1 year after transplantation 6) Adverse events graded by severity [21]
Study design	Both randomized controlled trials and non-randomized studies including case series with a minimum of 20 cases will be included. Case reports and studies that only focus on immunoglobulin use for de-sensitization purposes will be excluded

### Data extraction

Each eligible study will undergo a standardized data extraction process by two reviewers independently using a pre-designed form designed in Microsoft Excel (Microsoft Corporation, Seattle, WA, USA). Any discrepancies will be documented, discussed, and adjudicated by a third party. Prior to extraction of all study data, piloting of the form will first be conducted upon a small number of studies.

Information pertaining to study identification (author, year of publication, number and location of centers, funding, and journal); study design (type of study, sample size, eligibility criteria, and length of follow-up); aggregate patient characteristics (age, gender, duration of end organ damage prior to transplantation, type of transplantation, immunosuppressive regimen, comorbidities, onset of hypogammaglobulinemia, duration of hypogammaglobulinemia, treatment with immunoglobulin therapy either intravenously or subcutaneously, dose of immunoglobulin, and duration of treatment); and infection outcomes (the overall rate of infection as well as the rates of non-severe, severe, and serious/fatal infections) will be extracted. Hypogammaglobulinemia is defined as IgG level of less than 7 g/L. Severe hypogammaglobulinemia is defined as IgG level <4 g/L [4]. A serious/fatal infection will be considered to be any infection that required admission to intensive care unit or result in death. A severe infection will be considered any infection that required intravenous antimicrobial therapy and/or hospitalization. A non-severe infection will be considered any infection that does not meet criteria for severe or serious/fatal infection [16, 17]. If available, etiologic agent of infection will also be recorded as gram-positive, gram-negative, or anaerobic bacteria; *Candida* spp*.;* filamentous fungi; or viruses. Site of infection will be recorded as central nervous system, respiratory tract, gastrointestinal, genitourinary, skin, soft tissue, or blood stream infection related/non-related to catheter. Infection at other sites not specified above will be recorded as others. We will use individual study’s definitions of infectious outcomes if they do not align with the set definition above.

### Risk of bias assessment

Risk of bias in the randomized trials will be evaluated using the Cochrane Risk of Bias Assessment Tool [18]. Studies will be assessed based on the domains of randomization, generation of allocation sequence, allocation concealment, blinding, follow-up, and reporting. Non-randomized studies will be evaluated using the ACROBAT-NRSI [19] based on study design, confounding, reporting, and directness of evidence. Results from risk of bias assessments will be presented in their entirety. They will also be summarized narratively and may also inform sensitivity analyses. If there are sufficient numbers of trials (e.g., ≥ten studies), we will construct a funnel plot to assess for possible publication bias.

### Data analysis

For studies assessing the effect of immunoglobulin treatment, we will report the overall rates of infection, non-severe infection, severe infection, and serious/fatal infection per 100 patient-days in patients receiving immunoglobulin treatment for secondary hypogammaglobulinemia. We will also report stratified infection rates for patients with severe and non-severe hypogammaglobulinemia. Type of infection (e.g., bacterial, viral) and site of infection will also be reported. For studies assessing the effect of prophylactic immunoglobulin, we will report overall rates of infection and mortality, as well as adverse events.

We will compare overall rates of infection in the intervention and control (no IVIG) groups of each study and report the corresponding relative risk measures along with the corresponding with 95 % confidence interval. A relative risk of less than 1 will suggest a beneficial effect of intravenous immunoglobulin, while a relative risk of greater than 1 will suggest a harmful effect. Prior to conduct of meta-analyses, team members will review characteristics of the included studies and their patient populations such as type of study, outcome measures, and organ transplanted to establish the extent to which they appear homogeneous. The Cochrane Q/chi-square test and *I*^2^ statistic will also be calculated to evaluate heterogeneity. We will use *I*^2^ cutoff of ≥75 % to be considered as considerable heterogeneity [20]. Where meta-analysis is considered to be appropriate, we will pool data using a random effects model. Meta-analysis of non-randomized studies will be performed separately from meta-analysis of randomized trials. A systematic narrative synthesis will be provided with information presented in the text and tables to summarize and explain the characteristics and findings of the included studies. The narrative synthesis will explore the relationship and findings both within and between the included studies, in line with the guidance from the Centre for Reviews and Dissemination [15].

We will perform several a priori sensitivity analyses to understand the data and to identify any subpopulations that may benefit from the use of intravenous immunoglobulin. These analyses will include stratification of studies according to baseline immunoglobulin level (IgG ≤4, IgG >4 but <7, IgG ≥7 g/L), intent of immunoglobulin use (prophylaxis vs treatment), immunosuppressive regimen used (e.g., cyclosporin vs tacrolimus, induction vs no induction), and organ type (e.g., liver, kidney).

### Reporting of the review

We will report our findings in accordance with guidance of the PRISMA statement [15] and its checklist. A completed copy of the checklist will be provided in a supplement to the main report (Additional file 2).

## Discussion

In this systematic review, we will assess the treatment outcome of hypogammaglobulinemia post solid organ transplantation. We anticipate several challenges in this work. These will include the possibility of considerable study heterogeneity in terms of organ type, immunosuppressive regimen, study design, and outcomes reported. The availability of high-quality studies addressing this topic may also be limited. Studies may concentrate on a particular type of solid organ transplantation, in which case the data will be pooled and analyzed within the same type of transplantation. This may limit the generalizability to other types of solid organ transplantation but may provide for a better-focused interpretation.

Despite several anticipated challenges, our systematic review will provide important insights into the effects of immunoglobulin replacement therapy or prophylactic therapy in solid organ transplant recipients. The results may have a direct impact on current practice and practice guidelines. The data will also serve as a starting point for further research.

## Additional files

Additional file 1:
**Search strategy.** Serch terms and databases are outlined. (DOCX 15.2 kb) Additional file 2:
**PRISMA-P checklist.** Report of compliance with standard reporting guideline. (DOC 84 kb)

## Abbreviations

HLA: human leukocyte antigen
IgG: gamma-immunoglobulin
IVIG: intravenous immunoglobulin
SOT: solid organ transplantation
