# Supplementary material for: Clinical outcomes of immunoglobulin use in solid organ transplant recipients: protocol for a systematic review and meta-analysis
Source: Syst Rev. 2015 Nov 19;4:167. doi: 10.1186/s13643-015-0156-6 (PMC4653871; doi:10.1186/s13643-015-0156-6)
Supplement: Additional file 1: — Search strategy. Serch terms and databases are outlined. (DOCX 15.2 kb) [file 13643_2015_156_MOESM1_ESM.docx]

Database: Embase Classic+Embase <1950 to 2015 September>, Ovid MEDLINE(R) In-Process & Other Non-Indexed Citations and Ovid MEDLINE(R) <1950 to September>

--------------------------------------------------------------------------------

1 exp Organ Transplantation

2 exp Intestines/tr

3 ((organ or renal or kidney or pancreas or lung or heart or intestin$ or bowel) adj2 (transplant$ or graft$)).tw.

4 1 or 2 or 3

5 Agammaglobulinemia/

6 hypogammaglobulinemi$.tw.

7 Agammaglobulinemi$.tw.

8 immunoglobulin deficien$.tw.

9 or/5-8

10 4 and 9

11 exp Immunoglobulins/ and (exp Immunization, Passive/ or exp Administration, Intravenous/ or exp Injections, Subcutaneous/ or exp Infusions, Subcutaneous/)

12 Immunoglobulin$.tw.

13 Immune Globulin$.tw.

14 (ivig or (Intravenous adj5 IG) or (iv adj5 ig) or (iv adj5 igg)).tw.

15 or/11-14

16 10 and 15

17 16 use prmz

18 exp organ transplantation/

19 ((organ or renal or kidney or pancreas or lung or heart or intestin$ or bowel) adj2 (transplant$ or graft$)).tw.

20 18 or 19

21 exp immunoglobulin deficiency/

22 hypogammaglobulinemi$.tw.

23 Agammaglobulinemi$.tw.

24 immunoglobulin deficien$.tw.

25 or/21-24

26 20 and 25

27 exp immunoglobulin/iv, sc

28 immunoglobulin$.tw.

29 exp immunoglobulin/ and (intravenous drug administration/ or subcutaneous drug administration/ or passive immunization/)

30 immunoglobulin$.tw.

31 Immune Globulin$.tw.

32 (ivig or (Intravenous adj5 IG) or (iv adj5 ig) or (iv adj5 igg)).tw.

33 or/27-32

34 26 and 33

35 34 use emczd

36 17 or 35

37 remove duplicates from 36

38 37 use prmz

39 37 use emczd

Database: EBM Reviews - Cochrane Central Register of Controlled Trials <September 2015>

--------------------------------------------------------------------------------

1 exp Organ Transplantation/

2 exp Intestines/tr

3 ((organ or renal or kidney or pancreas or lung or heart or intestin$ or bowel) adj2 (transplant$ or graft$)).tw,hw.

4 1 or 2 or 3

5 Agammaglobulinemia/

6 hypogammaglobulinemi$.tw,hw.

7 Agammaglobulinemi$.tw,hw.

8 immunoglobulin deficien$.tw,hw.

9 or/5-8

10 4 and 9

11 exp Immunoglobulins/ and (exp Immunization, Passive/ or exp Administration, Intravenous/ or exp Injections, Subcutaneous/ or exp Infusions, Subcutaneous/)

12 Immunoglobulin$.tw,hw.

13 Immune Globulin$.tw,hw.

14 (ivig or (Intravenous adj5 IG) or (iv adj5 ig) or (iv adj5 igg)).tw,hw.

15 or/11-14

16 10 and 15
